# Mortality trends among people with hepatitis B and C: a population-based linkage study, 1993-2012

**DOI:** 10.1186/s12879-018-3110-0

**Published:** 2018-05-09

**Authors:** Maryam Alavi, Jason Grebely, Behzad Hajarizadeh, Janaki Amin, Sarah Larney, Matthew G. Law, Jacob George, Louisa Degenhardt, Gregory J. Dore

**Affiliations:** 1 Biostatistics and Databases Program, The Kirby Institute, UNSW Sydney, Wallace Wurth Building, UNSW, Sydney, NSW 2052 Australia; 2 Department of Health Systems and Populations, Macquarie University, Sydney, NSW Australia; 3 National Drug and Alcohol Research Centre, UNSW Sydney, Sydney, NSW Australia; 4 Storr Liver Centre, Westmead Millennium Institute, University of Sydney and Westmead Hospital, Westmead, NSW Australia

**Keywords:** HBV, HCV, Cause-specific mortality, Drug-related mortality, Liver-related mortality

## Abstract

**Background:**

This study evaluated cause-specific mortality trends including liver-related mortality among people with a hepatitis B virus (HBV) and hepatitis C virus (HCV) notification in New South Wales, Australia.

**Methods:**

Notifications 1993-2012 were linked to cause-specific mortality records 1993-2013.

**Results:**

Among 57,929 and 92,474 people with a HBV and HCV notification, 4.8% and 10.0% died since 1997. In early 2010s, 28% and 33% of HBV and HCV deaths were liver-related, 28% and 17% were cancer-related (excluding liver cancer), and 5% and 15% were drug-related, respectively. During 2002-2012, annual HBV-related liver death numbers were relatively stable (53 to 68), while HCV-related liver death numbers increased considerably (111 to 284). Age-standardised HBV-related liver mortality rates declined from 0.2 to 0.1 per 100 person-years (PY) (*P* < 0.001); however, HCV-related rates remained stable (0.2 to 0.3 per 100 PY, *P* = 0.619). In adjusted analyses, older age was the strongest predictor of liver-related mortality [birth earlier than 1945, HBV adjusted hazard ratio (aHR) 28.1, 95% CI 21.0, 37.5 and; HCV aHR 31.9, 95% CI 26.8, 37.9], followed by history of alcohol-use disorder (HBV aHR 7.0, 95% CI 5.5, 8.8 and; HCV aHR 8.3, 95% CI 7.6, 9.1).

**Conclusions:**

Declining HBV-related liver mortality rates and stable burden suggest an impact of improved antiviral therapy efficacy and uptake. In contrast, the impact of interferon-containing HCV treatment programs on liver-related mortality individual-level risk and population-level burden has been limited. These findings also highlight the importance of HBV/HCV public health interventions that incorporate increased antiviral therapy uptake, and action on health risk behaviors.

**Electronic supplementary material:**

The online version of this article (10.1186/s12879-018-3110-0) contains supplementary material, which is available to authorized users.

## Background

The global burden of chronic hepatitis B virus (HBV) and hepatitis C virus (HCV) infections is rising, with HBV and HCV-related deaths increasing from 0.8 to 1.4 million over the period 1990 to 2013 [1]. Ageing populations with chronic HBV and HCV and limited uptake of effective antiviral therapy are driving this burden. Therapeutic advances, particularly the advent of direct-acting antiviral (DAA) therapy for chronic HCV, provide optimism that major reductions in population-level liver-related mortality are achievable [2]. In fact, the World Health Organization (WHO) has recently set a target of a 65% reduction in HBV- and HCV-related mortality by 2030 [3]. In this context, ongoing surveillance of mortality trends is crucial to evaluate the efficacy of implementation of public health strategies against these infections.

Evaluation of mortality among people with HBV and HCV should ideally involve characterisation of cause-specific mortality. Co-morbidities are particularly prevalent among HCV populations, related to ongoing drug and alcohol use [4, 5], extrahepatic manifestations of HCV [6], or ageing-related chronic disorders [7]. Even within liver-related mortality there may be differential trends for decompensated cirrhosis (DC) and hepatocellular carcinoma (HCC) [8]. Characterisation of these mortality patterns before the rapid scale-up of DAA therapies is a public health priority, providing a foundation for the evaluation of the impact of new treatments on the disease burden of HCV.

Globally, Australia is among the few settings with established national surveillance systems that enable ongoing monitoring of all people notified with HBV and HCV infections, by linking notifications, hospitalisation, and mortality databases. The aim of this study was to assess cause-specific mortality trends and liver-related mortality risk factors among people with an HBV and HCV notification in New South Wales (NSW), Australia.

## Methods

### Study population and data sources

The study population consisted of all people recorded in the NSW Notifiable Conditions Information Management System (NCIMS) with an HBV and HCV notification. NSW NCIMS holds records of all individuals with positive HBV and HCV serology tests, notified of diagnoses via mandatory notification procedures, since 1991 [9]. Notifiable HBV and HCV cases require detection of HBV surface antigen or HBV DNA and anti-HCV antibody or HCV RNA, respectively.

People with HIV co-infection were identified through the National HIV Registry (NHR). NHR receives all notifications of HIV infection in Australia via mandatory notification procedures, since 1985 [10].

Deceased people were identified through the NSW Registry of Births, Deaths and Marriages (RBDM) and Cause of Death Unit Record File (COD URF). All deaths in NSW are registered within the NSW RBDM (since 1993) and coded causes are held within the COD URF. Since 1997, deaths are coded using the 10th revision of the International Classification of Diseases and Related Health Problems (ICD-10) [9].

Hospital admissions for alcohol-use disorder (AUD) and end-stage liver disease (ESLD) were identified through the NSW Admitted Patient Data Collection (APDC). Since 2001, NSW APDC holds inpatient admission information from all NSW hospitals, including diagnosis information coded at the time of discharge by ICD-10 [9]. People with a history of opioid substitution therapy (OST) were identified through the Pharmaceutical Drugs of Addiction System (PHDAS). PHDAS is used by the NSW Ministry of Health to issue authorities for applicant prescribers to prescribe drugs of addiction, including methadone (since 1985) and buprenorphine (since 2001) under the NSW Opioid Treatment Program [9].

### Data linkage

Data linkage occurred in two stages. First, NSW NCIMS HBV and HCV notifications were matched internally, to identify people with co-infection. Second, records were linked deterministically and probabilistically between the NSW NCIMS and NHR, and the NSW NCIMS and APDC, PHDAS, and RBDM and COD URF, respectively. Demographic details used for record linkages included full name, name codes (only for NHR), gender, date of birth, and address. NSW Centre for Health Record Linkage undertook all the linkages. Probabilistic linkage procedures had an error rate of 0.5% or less (i.e. false positive and false negative rates of 5/1000) [9].

### Study period

HBV and HCV notifications and mortality records were extracted for the study period between 1 January 1993 and 31 December 2012, 1 January 1993 and 31 December 2013, respectively.

### Study outcome

The primary outcome of interest was liver-related mortality, defined by multiple causes of death (i.e. coded in the underlying and/or contributing fields of a linked record, Additional file 1: Table S1). To improve the accuracy of this definition, contributing fields were included only if a prior ESLD-related hospitalisation was documented (defined by DC and/or HCC admissions, Additional file 1: Table S1). This definition was selected after considering a number of different classifications. Compared to those excluding secondary causes, a multiple-cause definition was believed to complement the routine description of liver-related mortality that would use only the underlying cause [11]. In the sensitivity analyses, trends of liver-related mortality were evaluated using three alternative definitions, including underlying and/or contributing causes of death (definition 2); underlying causes (definition 3); and underlying causes with prior ESLD admission (definition 4).

### Exclusion criteria

Records where date of death was prior to introduction of ICD-10 coding (calendar year 1997), and records where the date of HBV or HCV notification occurred after censoring were excluded.

### Statistical analysis

Among people with an HBV and HCV notification, cause-specific mortality numbers were first described by chapters of ICD-10. Liver-related mortality numbers were compared between four definitions, defined over several chapters of ICD-10, and ranging from the most conservative (definition 4) to the least conservative estimates (definition 2). Trends in cause-specific mortality (liver-, circulatory system-, drug-, and cancer-related, excluding liver cancer) numbers, incidence rates, and age-standardised incidence rates [per 100 person-years (PY)] were evaluated. The Australian Standard Population 2001 was used for standardisation. The strength of the association between risk factors and liver-related mortality was assessed by unadjusted and adjusted Cox proportional hazards regressions. Risk factors included birth cohort, gender, country of birth, calendar period of hepatitis notification, HBV/HCV/HIV co-infection, geographical area of residence at the time of hepatitis notification, history of AUD, and history of OST. Following unadjusted analyses, multivariable regressions were performed to evaluate factors associated with liver-related mortality, considering factors significant at the 0.20 level in the unadjusted models.

A multiple-cause definition was not used for non-liver-related causes of death (circulatory system-, drug-, and cancer-related); i.e. in all analyses, only the underlying field of a linked record was used to define these deaths (Additional file 1: Table S1). Other cause-related mortality was defined excluding ICD-10 codes used in the definitions of liver-, circulatory system-, drug, and cancer-related mortality (excluding liver cancer). AUD was the label used to define continued drinking despite adverse mental and physical consequences [12], and having at least one AUD-related hospital admission was referred to as history of AUD. AUD-related admissions were defined using the primary and/or secondary fields of linked hospitalisation records (Additional file 1: Table S1). In all analysis, having at least one episode of OST was referred to as history of OST.

To calculate all-cause and ICD-10 chapter-specific mortality numbers, all linked mortality records between 1997 and 2013 were included in analyses (including deaths that occurred within 6 months post the date of HCV notification). To calculate cause-specific mortality numbers and rates (liver-, circulatory system-, drug-, and cancer-related), and assess factors associated with liver-related mortality; linked mortality records between 2002 and 2013 were included in analyses. In all survival analyses, linked mortality records prior to 1 January 2002 were censored, given availability of hospitalisation data since 2001 (required to define liver-related mortality definition numbers one and four). In all survival analyses, people with a missing date of birth were excluded. Finally, in all survival analyses, observation time was defined to start 6 months post the date of HCV notification [13], and to end at whichever occurred first; death, or end of follow-up. Statistical analyses were carried out in STATA versions 12.

## Results

### Study population

During 1993-2012, 57,929 and 92,474 people in NSW had an HBV and HCV notification, respectively. Since 1997 (use of ICD-10 codes), 4.8% and 10.0% of the populations with an HBV and HCV notification died, respectively. Among those who had died, 42% and 52% were born during 1945-1965, 75% and 71% were male, 3% and 2% had HIV co-infection, 13% and 29% had a history of AUD, and 8% and 31% had a history of OST, respectively. Among people with an HBV notification, 17% of the deceased had HBV/HCV co-infection (Table 1).

**Table 1:** Demographic characteristics among people with an HBV and HCV notification, NSW 1993-2012, *n* = 150,403

Characteristics, n %	HBV, *n* = 57,929				HCV, *n* = 92,474			
	Alive		Deceased^a^		Alive		Deceased^a^	
	*n* = 55,147	%	*n* = 2782	%	*n* = 83,267	%	*n* = 9207	%
Birth cohort^b^								
≥ 1965	31,000	56	423	15	43,919	53	2253	24
1945-1964	20,595	37	1166	42	35,964	43	4795	52
≤ 1944	3541	6	1193	43	3359	4	2159	23
Male gender^b^	29,949	55	2074	75	51,062	62	6507	71
Country of birth^b^								
Australia	5406	20	546	30	34,263	78	4030	71
Asia-Pacific	16,692	63	817	45	4037	9	490	9
Middle East and North Africa	2029	8	85	5	1263	3	198	4
Africa, excluding North Africa	577	2	14	1	191	< 1	15	< 1
Europe	1782	7	322	18	3512	8	884	16
Americas	134	1	11	1	415	1	39	1
Year of viral hepatitis notification								
≤ 2000	24,696	45	1634	59	43,067	52	6014	65
2001-2006	16,514	30	782	28	23,250	28	2229	24
2007-2012	13,937	25	366	13	16,950	20	964	10
HBV/HCV co-infection^c^	3232	6	480	17	–	–	–	–
HIV co-infection	276	1	72	3	812	1	153	2
HBV/HCV/HIV co-infection^c^	50	< 1	14	< 1	–	–	–	–
Area of residence at the time of viral hepatitis notification^b,d^								
Rural	5342	10	440	16	28,610	35	2963	32
Outer metropolitan	25,068	46	1245	45	26,608	33	3167	35
Metropolitan	24,183	44	1082	39	26,331	32	3025	33
History of alcohol-use disorder	1566	3	374	13	12,855	15	2652	29
History of OST	1442	3	225	8	24,427	29	2813	31

^a^Mortality numbers included during the ICD-10 era, 1997-2013, ^b^among people with available information: *n* = 36 and *n* = 25 had missing date of birth among those with an HBV and HCV notifications, respectively; *n* = 500 and *n* = 341 had missing gender among those with an HBV and HCV notifications, respectively; *n* = 29,514 and *n* = 43,137had missing country of birth among those with an HBV and HCV notifications, respectively and; *n* = 569 and *n* = 1718 had missing area of residence at the time of HBV and HCV notifications, respectively, ^c^for description of baseline characteristics, HBV/HCV and HBV/HCV/HIV co-infection cases were only included among people with an HBV notification, ^d^geographical area of residence was defined by the 2011 local health district boundaries in NSW, including eight areas covering metropolitan regions and seven covering rural and regional locations

Infectious and parasitic diseases comprised 10% of HBV- and HCV-related deaths. Among people with an HBV notification, neoplasms were the leading cause of mortality (44%), followed by diseases of the circulatory system (17%). External causes of death and neoplasms were the first and second most frequently recorded causes of HCV-related mortality (25% and 24%, respectively) (Additional file 1: Table S2).

### Liver-related mortality, definitions

**Fig. 1:**
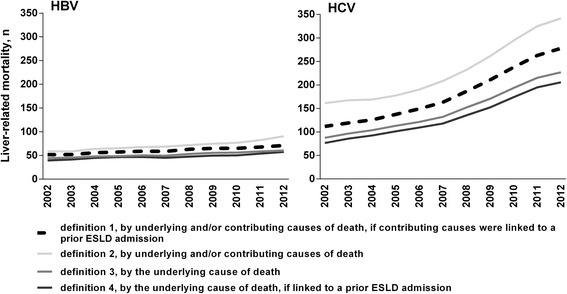
Liver-related mortality numbers and definitions among people with an HBV and HCV notification, NSW 1993-2012, *n* = 150,403

By the most conservative definition (definition 4), during 2002-2012, HBV- and HCV-related liver mortality numbers increased from 39 to 56 and 74 to 208, respectively. Defined by multiple causes of death, during 2002-2012, HBV- and HCV-related liver mortality numbers rose from 53 to 68 and 111 to 284, respectively (Fig. 1).

### Cause-specific mortality, age-specific and age-standardized rates

**Fig. 2:**
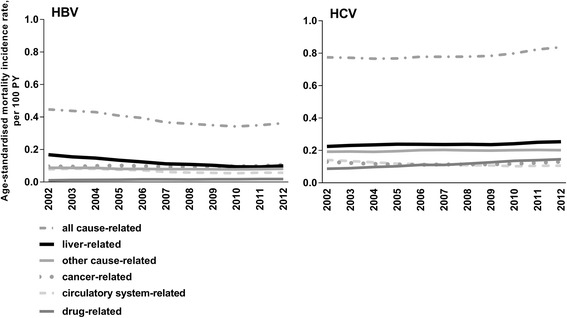
Age-standardised mortality incidence rates among people with an HBV and HCV notification, NSW 1993-2012, *n* = 150,403

**Fig. 3:**
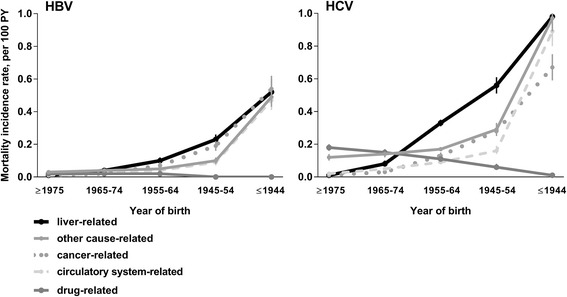
Cause-specific mortality incidence rates among people with an HBV and HCV notification, NSW 1993-2012, by year of birth, *n* = 150,403

During 2002-2012, among people with an HBV notification, age-standardised rates of liver-related mortality (*n* = 582) declined from 0.2 to 0.1 per 100 PY (*P* < 0.001) (Fig. 2). Incidence rates of liver-, circulatory system- (*n* = 340), and cancer-related deaths (*n* = 502) increased with increasing age, noticeably among people born during 1955-1964 and earlier (Fig. 3).

During 2002-2012, among people with an HCV notification, age-standardised rates of liver-related mortality (*n* = 2215) remained stable, 0.2 to 0.3 per 100 PY, *P* = 0.619. Age-standardised rates of circulatory system-related mortality (*n* = 985) lowered from 0.2 to 0.1 per 100 PY, *P* = 0.026. However, rates of drug-related mortality (*n* = 1110) increased during this period, from 0.1 to 0.2 per 100 PY, *P* < 0.001 (Fig. 2). Incidence rates of liver- and cancer-related deaths (*n* = 1072) increased with increasing age, markedly among people born during 1955-1964 and earlier. However, drug-related mortality rates were highest among people born in 1975 or later, and lower among older age groups (Fig. 3).

### Liver-related mortality, associated factors

In unadjusted analysis, HBV-related liver mortality was associated with older age, male gender, HCV co-infection, HIV co-infection, history of AUD and history of OST. People born in Asia-Pacific and those residing in outer metropolitan and metropolitan areas of NSW at the time of HBV notification had reduced liver-related mortality risk (Additional file 1: Table S3). In adjusted analysis, HBV-related liver mortality was associated with older age (birth earlier than 1945, and during 1945-1965), male gender, more recent periods of HBV notification years (2001-2006, and 2007-2012), HCV co-infection, HIV co-infection, and history of AUD (Table 2).

**Table 2:** Adjusted analysis of factors associated with liver-related mortality among people with an HBV and HCV notification, NSW 1993-2012, *n* = 150,403

Characteristics, n %	HBV					HCV				
	Deceased^a^		aHR^b^	95% CI	*P*	Deceased^a^		aHR^b^	95% CI	*P*
	*n* = 582	%				*n* = 2215	%			
Birth cohort										
≥ 1965	66	< 1	1.00	–	–	247	1	1.00	–	–
1945-1964	304	1	6.19	4.72, 8.11	< 0.001	1542	4	7.33	6.39, 8.40	< 0.001
≤ 1944	212	5	28.07	21.02, 37.47	< 0.001	426	9	31.88	26.83, 37.87	< 0.001
Gender										
Female	102	< 1	1.00	–	–	559	2	1.00	–	–
Male	477	2	2.65	2.13, 3.30	< 0.001	1654	3	1.47	1.33, 1.62	< 0.001
Missing	3	1	1.44	0.45, 4.54	0.539	2	1	0.41	0.10, 1.64	0.208
Country of birth^c^										
Australia	109	2	1.00	–	–	1024	3	1.00	–	–
Asia-Pacific	148	1	0.89	0.67, 1.19	0.425	130	3	1.07	0.88, 1.29	0.493
Other	76	2	0.91	0.67, 1.25	0.580	311	5	1.43	1.25, 1.63	< 0.001
Missing	249	1	0.86	0.66, 1.12	0.268	750	2	0.88	0.79, 0.97	0.015
Year of notification										
≤ 2000	316	1	1.00	–	–	1347	3	1.00	–	–
2001-2006	206	1	1.26	1.01, 1.58	0.041	627	2	1.48	1.32, 1.67	< 0.001
2007-2012	60	< 1	1.44	1.01, 2.06	0.045	241	1	2.98	2.47, 3.60	< 0.001
HBV/HCV co-infection										
No	461	1	1.00	–	–	2094	2	1.00	–	–
Yes	121	3	2.63	2.03, 3.41	< 0.001	121	3	1.53	1.28, 1.85	< 0.001
HIV co-infection										
No	572	1	1.00	–	–	2186	2	1.00	–	–
Yes	10	3	3.46	1.83, 6.55	< 0.001	29	3	1.78	1.23, 2.57	0.002
Area of residence at the time of notification										
Rural	92	2	1.00	–	–	–	–	–	–	–
Outer metropolitan	262	1	1.02	0.78, 1.32	0.899	–	–	–	–	–
Metropolitan	227	1	0.84	0.65, 1.10	0.207	–	–	–	–	–
Missing	1	< 1	0.21	0.03, 1.53	0.124	–	–	–	–	–
History of alcohol-use disorder										
No	448	1	1.00	–	–	1050	1	1.00	–	–
Yes	134	7	6.98	5.54, 8.78	< 0.001	1165	7	8.30	7.56, 9.10	< 0.001
History of OST^d^										
No	538	1	1.00	–	–	1675	3	1.00	–	–
Yes	44	3	0.84	0.57, 1.25	0.391	540	2	0.92	0.82, 1.03	0.150

^a^Records included 2002-2013, ^b^adjusted hazard ratio, ^c^majority of countries included in the other category were European (HBV n = 50, HCV *n* = 229). Non-European countries were included in the other category given the small numbers (HBV *n* = 26, HCV *n* = 72), ^d^included as a time-dependent variable

In unadjusted analysis, HCV-related liver mortality was associated with older age, male gender, more recent periods of HCV notification, HBV co-infection, HIV co-infection and history of AUD. People with a history of OST had reduced liver-related mortality risk (Additional file 1: Table S4). In adjusted analysis, HCV-related liver mortality was associated with older age (birth earlier than 1945, and during 1945-1965), male gender, recent periods of HCV notification (2001-2006, and 2007-2012), HBV co-infection, HIV co-infection, and history of AUD (Table 2).

### Cause-specific mortality, temporal trends

**Fig. 4:**
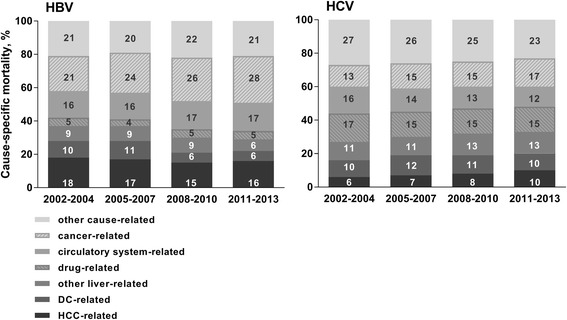
Percentage of deaths due to different underlying causes among people with an HBV and HCV notification, NSW 1993-2012, *n* = 150,403

Among people with an HBV notification, DC-related mortality decreased from 10% of all causes in 2002-2004 to 6% in 2011-2013 (*P* < 0.001); however, proportions of HCC-related mortality remained stable during this period (18% and 16% of all causes, *P* = 0.692). In the early 2010s, other cancers and diseases of the circulatory system comprised 28% and 17% of all deaths, respectively (Fig. 4).

Among people with an HCV notification, DC-related mortality comprised 10% of all causes in 2002-2004 and 2011-2013 (*P* = 0.277); however, HCC-related mortality increased from 6% to 10% during this period (*P* < 0.001). In the early 2010s, other cancers and drug-related causes comprised 17% and 15% of all deaths, respectively (Fig. 4).

### Other cause-related mortality

Among people with an HBV notification, there were 620 other cause-related deaths. The majority of these deaths occurred among people born in 1954 or earlier (60%, *n* = 370) and were most frequently due to infectious and parasitic diseases (17%, 63 of 370). Among people with an HCV notification, there were 2298 other cause-related deaths. The majority of these deaths occurred among people born in 1955 or later (61%, *n* = 1397) and were most frequently due to external causes (59%, *n* = 821), including intentional self-harm by hanging, strangulation, and suffocation (28%, 232 of 821), other self-harm (16%, 134 of 821), and transport accidents (20%, 165 of 821) (Additional file 1: Table S5).

## Discussion

This study demonstrated opposing trends in liver-related mortality among people with an HBV and HCV notification in NSW, Australia. During 2002-2012, among people with HBV age-standardised risk of liver mortality has reduced significantly, while the population-level burden of liver mortality (total deaths per year) has remained relatively stable. By contrast, among people with HCV age-standardised risk of liver mortality has remained relatively stable, while population-level burden has markedly increased. The potential impact of improving HBV antiviral therapy since the mid-2000s is encouraging; however, these trends underline the relatively limited impact of interferon-based HCV treatment. Non-liver-related causes comprised the majority of HBV and HCV deaths throughout the study period, highlighting the need for a comprehensive strategy for reducing morbidity and mortality among people with HBV and HCV. Mandatory HBV and HCV notifications, availability of this data for research, and the capacity for regular linkages to other routinely collected administrative databases provides the opportunity for ongoing evaluation of HBV and HCV public health strategies, including the introduction of government-subsidized broad access to interferon-free DAA HCV treatment in Australia from 2016.

Among people with an HBV notification, proportions of DC-related mortality significantly declined between early 2000s and early 2010s; however, HCC-related mortality remained stable during this period. Improved HBV therapies have been shown to reduce the risks of hepatic events, HCC, liver-related, and all-cause mortality, particularly among people with cirrhosis [14]. However, current regimens cannot completely prevent HCC, and regular surveillance is still required in at-risk groups (predominantly those with cirrhosis) even when HBV DNA is undetectable [15]. In Australia, the number of people receiving HBV treatment has increased in recent years, from 8500 in 2006 to 11,000 in 2012 [16, 17]; however, substantial gaps remain in the HBV cascade of care, given that up to two-thirds of eligible individuals are not receiving therapy [17]. The potential to further reduce the mortality burden of HBV infection through enhanced diagnosis and treatment uptake is considerable.

Among people with an HCV notification, proportions of DC-related mortality were stable between early 2000s and early 2010s, while HCC-related mortality increased in this period. Despite improvements in HCV antiviral therapy, small numbers of individuals were treated during 2000s-early 2010s, and treatment outcomes were sub-optimal [18]. Moving forward, a combination of enhanced treatment efficacy and increased treatment uptake is expected to have a greater impact on HCV-related mortality [2]. In Australia, access to highly effective interferon-free DAA therapies has been provided via government subsidy from March 2016, regardless of disease stage or drug and alcohol use. This has enabled a rapid initial uptake of DAA therapy [19], with the potential to reduce liver-related mortality. Importantly, entry into HCV care through diverse models including primary care and drug and alcohol services could improve engagement and retention along the HCV care cascade and optimise co-morbidity management [20].

More recent notification periods (2001-2006, 2007-2012) were associated with increased risk of HBV and HCV liver-related mortality, an association that is likely to be driven by age (Additional file 1: Table S6). Other factors associated with liver-related mortality included older age, male gender, HBV/HCV/HIV co-infection, and history of AUD. Given the accelerated progression to advanced liver disease [21–23], where appropriate, these characteristics could be used to constitute high-risk groups that may need to be prioritised in the era of improved antiviral therapies.

Drug-related mortality remained a major cause of death among people with an HCV notification during 2002-2012, which is not surprising given that the majority of HCV transmissions are among people who inject drugs. In Australia, this period was characterised by shifting heroin and methamphetamine markets, changing patterns of drug use (including poly drug use), and increases in extra-medical opioid use [24–27]. In this context, the rising individual-level risk of drug-related mortality underlines the need for broader access to harm reduction and treatment programs that are responsive to the changing needs of people who use drugs. In addition to mortality from direct effects of drug use, this study showed a substantial number of deaths among people with HCV are due to suicide and accidental injuries. These results were sobering, in highlighting the importance of a multidisciplinary response to HCV that encompasses not only provision of antiviral therapy, but also strategies to improve mental health- and substance use-related outcomes [28].

As populations with HBV and HCV age, mortality from non-communicable diseases increases in prominence [7]. Circulatory system-related mortality remained a major cause of HBV and HCV deaths in early 2010s; however, declines in its individual-level risk (particularly among people with an HCV notification) is consistent with trends in other high income countries [29]. Nevertheless, these trends are dynamic, given changes in the prevalence of risk factors and uncertainties about the impact of enhanced HCV treatment uptake [6, 30], and should be closely monitored.

This study has several limitations. First, in Australia, HBV notifications are based on evidence of chronic infection. However, the number of people with active HBV replication could not be evaluated. Second, HCV diagnosis for surveillance does not necessitate HCV RNA confirmation, and is commonly based on anti-HCV antibody detection. Therefore, an estimated 25% of people with an HCV notification would have spontaneously cleared their HCV infection. Nevertheless, these limitations are not thought to have a significant impact on the findings of this study, given fixed surveillance definition and systems in NSW during the study period. Third, in the presence of multiple chronic diseases that are each potentially fatal, the decision about selecting the underlying cause of death can be subjective; however, where possible (i.e. liver-related mortality), a multiple cause definition was used to include important cause information that might have been overlooked otherwise [11]. Fourth, in evaluation of the association between risk factors and liver-related mortality, a sensitivity analysis was performed using Fine and Gray regression, to account for competing risks. Period of HBV notification was not associated with liver-related mortality in the adjusted Fine and Gray model. This difference could be due to the potential impact of higher numbers of deaths among older people who had an HBV notification in recent time periods, and inclusion of these deaths in the risk set of a competing risk framework. Fifth, using administrative data to assess risk factors for HBV- and HCV-related liver mortality has clear limitations, including lack of sociodemographic and health risk information such as smoking, poor diet, physical inactivity, mental health problems, and low income. Sixth, DC-related hospitalisation and mortality were defined by a limited number of conditions that appear to be strong indicators of the decompensated stage of cirrhosis; however, this definition has not been validated against the clinical diagnosis of DC, and requires further validation studies. Finally, given lack of individual-level treatment data, in the analyses of factors associated with liver-related mortality, it was not possible to determine which associated factors were proxies for not receiving antiviral therapy. During the era of interferon-containing HCV treatments, factors including older age, advanced liver disease, and history of alcohol use could have been associated with lower HCV treatment uptake, given poorer response rates [31].

## Conclusions

In conclusion, this population-level data linkage study provides evidence for contrasting trends in HBV- and HCV-related liver mortality in NSW, Australia, a possible result of differences in antiviral therapy efficacy and uptake. These findings also suggest action against health risk behaviours should form a strong component of HBV and HCV public health strategies. Use of administrative databases for surveillance, particularly with the addition of individual-level antiviral treatment data will be a valuable tool for public health research and evaluation of improved strategies for viral hepatitis diagnosis and treatment in the future.

## Additional file

Additional file 1: **Table S1.** ICD-10 codes used to define cause-specific mortality and hospital admissions among people with an HBV and HCV notification, NSW 1993-2012, *n* = 150,403. **Table S2.** Cause-specific mortality among people with an HBV and HCV notification, NSW 1993-2012, by ICD-10 chapter, n = 150,403. **Table S3.** Unadjusted analysis of factors associated with liver-related mortality among people with an HBV notification, NSW 1993-2012, *n* = 57,929. **Table S4.** Unadjusted analysis of factors associated with liver-related mortality among people with an HCV notification, NSW 1993-2012, *n* = 96,250. **Table S5.** Other cause-related mortality among people with an HBV and HCV notification, NSW 1993-2012, by ICD-10 chapter and year of birth, *n* = 150,403. **Table S6.** Adjusted analysis of factors associated with liver-related mortality among people with an HBV and HCV notification, NSW 1993-2012, *n* = 150,403. (DOCX 50 kb)

## Abbreviations

APDC: Admitted patient data collection
AUD: Alcohol-use disorder
COD URF: Cause of death unit record file
DAA: Direct-acting antiviral
DC: Decompensated cirrhosis
ESLD: End-stage liver disease
HBV: Hepatitis B virus
HCC: Hepatocellular carcinoma
HCV: Hepatitis C virus
ICD-10: International classification of diseases and related health problems
NCIMS: Information management system
NHR: National HIV Registry
NSW: New South Wales
OST: Opioid substitution therapy
PHDAS: Pharmaceutical drugs of addiction system
RBDM: Registry of births, deaths and marriages
WHO: World Health Organization
