# Supplementary material for: Mortality trends among people with hepatitis B and C: a population-based linkage study, 1993-2012
Source: BMC Infect Dis. 2018 May 9;18:215. doi: 10.1186/s12879-018-3110-0 (PMC5944091; doi:10.1186/s12879-018-3110-0)
Supplement: Supplementary file 1 — Table S1. ICD-10 codes used to define cause-specific mortality and hospital admissions among people with an HBV and HCV notification, NSW 1993-2012, n = 150,403. Table S2. Cause-specific mortality among people with an HBV and HCV notification, NSW 1993-2012, by ICD-10 chapter, n = 150,403. Table S3. Unadjusted analysis of factors associated with liver-related mortality among people with an HBV notification, NSW 1993-2012, n = 57,929. Table S4. Unadjusted analysis of factors associated with liver-related mortality among people with an HCV notification, NSW 1993-2012, n = 96,250. Table S5. Other cause-related mortality among people with an HBV and HCV notification, NSW 1993-2012, by ICD-10 chapter and year of birth, n = 150,403. Table S6. Adjusted analysis of factors associated with liver-related mortality among people with an HBV and HCV notification, NSW 1993-2012, n = 150,403. (DOCX 50 kb) [file 12879_2018_3110_MOESM1_ESM.docx]

**Additional file 1**

**Table S1. ICD-10 codes used to define cause-specific mortality and hospital admissions among people with an HBV and HCV notification, NSW 1993-2012, n=150,403**

| **Cause-specific mortality and hospital admissions** | **ICD-10 codes** |
| --- | --- |
| **Mortality** |  |
| Liver-related^α^ |  |
| viral hepatitis, including acute, chronic, and unspecified viral hepatitis | B15-B19 |
| sequelae of viral hepatitis | B94.2 |
| primary liver cancer | C22 |
| alcoholic liver disease | K70 |
| non-alcoholic liver disease |  |
| toxic liver disease | K71 |
| hepatic failure and hepatic encephalopathy, , not elsewhere classified | K72 |
| chronic hepatitis, not elsewhere classified | K73 |
| liver fibrosis and cirrhosis of liver | K74 |
| other inflammatory liver disease | K75 |
| other diseases of liver and liver disorders in diseases classified elsewhere | K76, K77 |
| ascites | R18 |
| bleeding oesophageal varices | I85.0 |
| Circulatory system-related**^β^** |  |
| diseases of the circulatory system | I00-99 |
| Drug-related**^β^** |  |
| mental and behavioural disorders due to psychoactive substance use  (excluding alcohol, tobacco and volatile solvents, e.g. petrol) | F11-16 and F19 |
| abuse of non-dependence-producing substances | F55 |
| accidental poisoning by drugs | X40-44 |
| drug-related intentional self-harm | X60-64 |
| drug-related assault | X85 |
| deaths of undetermined intent by drugs, medicaments and biologicals | Y10-14 |
| Cancer-related**^β^** |  |
| neoplasms | C00-D48, excluding C22 |
| Other cause-related**^β^** |  |
| **Hospital admissions^γ^** | Codes not mentioned above |
| Alcohol-use disorder |  |
| alcohol-induced Pseudo-Cushing's syndrome | E24.4 |
| mental and behavioural disorders due to use of alcohol | F10 |
| degeneration of nervous system due to alcohol | G31.2 |
| alcohol polyneuropathy | G62.1 |
| alcoholic cardiomyopathy | I42.6 |
| alcohol myopathy | G72.1 |
| alcohol rehabilitation | Z50.2 |
| alcohol abuse counselling and surveillance | Z71.4 |
| End-stage liver disease |  |
| Decompensated cirrhosis |  |
| ascites | R18 |
| bleeding oesophageal varices | I85.0 and I98.3 |
| chronic hepatic failure (including hepatic encephalopathy) | K72.1 and K72.9 |
| alcoholic hepatic failure | K70.4 |
| hepatorenal syndrome | K76.7 |
| Hepatocellular carcinoma |  |
| hepatocellular carcinoma | C22.0 |

^α^defined by multiple causes of death, ^β^defined by the underlying cause of death, **^γ^**all causes defined by the primary and/or secondary causes of hospital admission

**Table S2. Cause-specific mortality among people with an HBV and HCV notification, NSW 1993-2012, by ICD-10 chapter, n=150,403**

| **ICD chapter, n %** | **Chapter description** | **HBV^α,β^**  **n=2,782** | **%** | **HCV^α,β^**  **n=9,207** | **%** |
| --- | --- | --- | --- | --- | --- |
| A00-B99 | Infectious and parasitic diseases | 280 | 10 | 960 | 10 |
| C00-D48 | Neoplasms | 1,236 | 44 | 2,181 | 24 |
| D50-D89 | Blood and blood-forming organs/immune mechanism | 14 | 1 | 41 | <1 |
| E00-E90 | Endocrine, nutritional, and metabolic diseases | 76 | 3 | 195 | 2 |
| F00-F99 | Mental and behavioural disorders | 70 | 3 | 577 | 6 |
| G00-G99 | Diseases of the nervous system | 39 | 1 | 142 | 2 |
| I00-I99 | Diseases of the circulatory system | 470 | 17 | 1,341 | 15 |
| J00-J99 | Diseases of the respiratory system | 87 | 3 | 360 | 4 |
| K00-K93 | Diseases of the digestive system | 142 | 5 | 848 | 9 |
| L00-L99 | Disease of the skin and subcutaneous tissue | 0 | - | 27 | <1 |
| M00-M99 | Diseases of the musculoskeletal and connective tissue | 12 | <1 | 36 | <1 |
| N00-N99 | Diseases of the genitourinary system | 51 | 2 | 148 | 2 |
| O00-Q99 | Pregnancy/perinatal/congenital conditions | 9 | <1 | 22 | <1 |
| R00-R99 | Symptoms and signs not elsewhere classified | 19 | 1 | 73 | 1 |
| V00-Y98 | External causes of mortality | 277 | 10 | 2,256 | 25 |

^α^mortality numbers included during the ICD-10 era, 1997-2013, ^β^for description of baseline characteristics, HBV/HCV and HBV/HCV/HIV co-infection cases were only included among people with an HBV notification

**Table S3. Unadjusted analysis of factors associated with liver-related mortality among people with an HBV notification, NSW 1993-2012, n=57,929**

|  | **Deceased^α^** |  | **HR^β^** | **95% CI** | ***P*** |
| --- | --- | --- | --- | --- | --- |
| **Characteristics, n %** | **n=582** | **%** |  |  |  |
| Birth cohort |  |  |  |  |  |
| ≥1965 | 66 | <1 | 1.00 | - | - |
| 1945-1964 | 304 | 1 | 6.27 | 4.80, 8.20 | <0.001 |
| ≤1944 | 212 | 5 | 23.10 | 17.49, 30.50 | <0.001 |
| Gender |  |  |  |  |  |
| Female | 102 | <1 | 1.00 | - | - |
| Male | 477 | 2 | 3.81 | 3.08, 4.72 | <0.001 |
| Missing | 3 | 1 | 1.64 | 0.52, 5.17 | 0.399 |
| Country of birth^γ^ |  |  |  |  |  |
| Australia | 109 | 2 | 1.00 | - | - |
| Asia-Pacific | 148 | 1 | 0.46 | 0.36, 0.58 | <0.001 |
| Other | 76 | 2 | 0.87 | 0.65, 1.17 | 0.359 |
| Missing | 249 | 1 | 0.45 | 0.36, 0.56 | <0.001 |
| Year of HBV notification |  |  |  |  |  |
| ≤2000 | 316 | 1 | 1.00 | - | - |
| 2001-2006 | 206 | 1 | 1.03 | 0.82, 1.28 | 0.819 |
| 2007-2012 | 60 | <1 | 0.79 | 0.56, 1.13 | 0.200 |
| HCV co-infection |  |  |  |  |  |
| No | 461 | 1 | 1.00 | - | - |
| Yes | 121 | 3 | 3.93 | 3.22, 4.80 | <0.001 |
| HIV co-infection |  |  |  |  |  |
| No | 572 | 1 | 1.00 | - | - |
| Yes | 10 | 3 | 3.47 | 1.86, 6.48 | <0.001 |
| HCV/HIV co-infection^δ^ |  |  |  |  |  |
| No | 581 | 1 | 1.00 | - | - |
| Yes | 1 | 1 | 2.25 | 0.32, 16.00 | 0.418 |
| Area of residence at the time of notification |  |  |  |  |  |
| Rural | 92 | 2 | 1.00 | - | - |
| Outer metropolitan | 262 | 1 | 0.60 | 0.47, 0.76 | <0.001 |
| Metropolitan | 227 | 1 | 0.55 | 0.43, 0.70 | <0.001 |
| Missing | 1 | <1 | 0.13 | 0.02, 0.97 | 0.046 |
| History of alcohol-use disorder |  |  |  |  |  |
| No | 448 | 1 | 1.00 | - | - |
| Yes | 134 | 7 | 9.52 | 7.84, 11.55 | <0.001 |
| History of OST^ε^ |  |  |  |  |  |
| No | 538 | 1 | 1.00 | - | - |
| Yes | 44 | 3 | 2.75 | 2.02, 3.73 | <0.001 |

^α^records included 2002-2013 ^β^hazard ratio, ^γ^majority of countries included in the other category were European (n=50). Non-European countries were included in the other category given the small numbers (n=26), ^δ^not considered for inclusion in the adjusted model, ^ε^included as a time-dependent variable

**Table S4. Unadjusted analysis of factors associated with liver-related mortality among people with an HCV notification, NSW 1993-2012, n=96,250**

|  | **Deceased^α^** |  | **HR^β^** | **95% CI** | ***P*** |
| --- | --- | --- | --- | --- | --- |
| **Characteristics, n %** | **n=2,215** | **%** |  |  |  |
| Birth cohort |  |  |  |  |  |
| ≥1965 | 247 | 1 | 1.00 | - | - |
| 1945-1964 | 1,542 | 4 | 6.69 | 5.85, 7.66 | <0.001 |
| ≤1944 | 426 | 9 | 17.16 | 14.67, 20.08 | <0.001 |
| Gender |  |  |  |  |  |
| Female | 559 | 2 | 1.00 | - | - |
| Male | 1,654 | 3 | 1.81 | 1.65, 2.00 | <0.001 |
| Missing | 2 | 1 | 0.33 | 0.08, 1.31 | 0.114 |
| Country of birth^γ^ |  |  |  |  |  |
| Australia | 1,024 | 3 | 1.00 | - | - |
| Asia-Pacific | 130 | 3 | 1.05 | 0.87, 1.25 | 0.636 |
| Other | 311 | 5 | 1.80 | 1.58, 2.04 | <0.001 |
| Missing | 750 | 2 | 0.59 | 0.53, 0.64 | <0.001 |
| Year of HCV notification |  |  |  |  |  |
| ≤2000 | 1,347 | 3 | 1.00 | - | - |
| 2001-2006 | 627 | 2 | 1.37 | 1.22, 1.54 | <0.001 |
| 2007-2012 | 241 | 1 | 2.31 | 1.92, 2.78 | <0.001 |
| HBV co-infection |  |  |  |  |  |
| No | 2,094 | 2 | 1.00 | - | - |
| Yes | 121 | 3 | 1.54 | 1.28, 1.85 | <0.001 |
| HIV co-infection |  |  |  |  |  |
| No | 2,186 | 2 | 1.00 | - | - |
| Yes | 29 | 3 | 1.60 | 1.11, 2.31 | 0.012 |
| HBV/HIV co-infection^δ^ |  |  |  |  |  |
| No | 2,214 | 2 | 1.00 | - | - |
| Yes | 1 | 2 | 1.07 | 0.15, 7.61 | 0.945 |
| Area of residence at the time of HCV notification^δ^ |  |  |  |  |  |
| Rural | 724 | 2 | 1.00 | - | - |
| Outer metropolitan | 746 | 2 | 1.05 | 0.95, 1.16 | 0.340 |
| Metropolitan | 735 | 2 | 1.02 | 0.92, 1.13 | 0.654 |
| Missing | 10 | 1 | 0.44 | 0.23, 0.82 | 0.009 |
| History of alcohol-use disorder |  |  |  |  |  |
| No | 1,050 | 1 | 1.00 | - | - |
| Yes | 1,165 | 7 | 6.11 | 5.62, 6.65 | <0.001 |
| History of OST^ε^ |  |  |  |  |  |
| No | 1,675 | 3 | 1.00 | - | - |
| Yes | 540 | 2 | 0.70 | 0.63, 0.77 | <0.001 |

^α^records included 2002-2013, ^β^hazard ratio, ^γ^majority of countries included in the other category were European (n=229). Non-European countries were included in the other category given the small numbers (n=72), ^δ^not considered for inclusion in the adjusted model, ^ε^included as a time-dependent variable

**Table S5. Other cause-related mortality among people with an HBV and HCV notification, NSW 1993-2012, by ICD-10 chapter and year of birth, n=150,403**

|  |  | **HBV^α,β^**  **n=620** | | | | | | | | | | **HCV^α,β^**  **n=2,298** | | | | | | | | | |
| --- | --- | --- | --- | --- | --- | --- | --- | --- | --- | --- | --- | --- | --- | --- | --- | --- | --- | --- | --- | --- | --- |
| **ICD chapter, n %** | **Chapter description** | **≥1975** | **%** | **65-74** | **%** | **55-64** | **%** | **45-54** | **%** | **≤1944** | **%** | **≥1975** | **%** | **65-74** | **%** | **55-64** | **%** | **45-54** | **%** | **≤1944** | **%** |
| A00-B99 | Infectious and parasitic diseases | 4 | 8 | 16 | 19 | 25 | 22 | 23 | 23 | 40 | 15 | 7 | 3 | 27 | 6 | 58 | 8 | 29 | 9 | 47 | 8 |
| C00-D48 | Neoplasms | 0 | - | 0 | - | 0 | - | 0 | - | 0 | - | 0 | - | 0 | - | 0 | - | 0 | - | 0 | - |
| D50-D89 | Blood and blood-forming organs/immune mechanism | 1 | 2 | 0 | - | 3 | 3 | 2 | 2 | 5 | 2 | 0 | - | 3 | 1 | 11 | 2 | 13 | 4 | 7 | 1 |
| E00-E90 | Endocrine, nutritional, and metabolic diseases | 3 | 6 | 2 | 2 | 8 | 7 | 12 | 12 | 42 | 16 | 9 | 4 | 14 | 3 | 41 | 6 | 28 | 8 | 87 | 15 |
| F00-F99 | Mental and behavioural disorders | 0 | - | 3 | 4 | 5 | 4 | 1 | 1 | 24 | 9 | 5 | 2 | 14 | 3 | 43 | 6 | 14 | 4 | 35 | 6 |
| G00-G99 | Diseases of the nervous system | 1 | 2 | 4 | 5 | 6 | 5 | 8 | 8 | 20 | 7 | 6 | 3 | 21 | 4 | 46 | 7 | 15 | 5 | 42 | 7 |
| I00-I99 | Diseases of the circulatory system | 0 | - | 0 | - | 0 | - | 0 | - | 0 | - | 0 | - | 0 | - | 0 | - | 0 | - | 0 | - |
| J00-J99 | Diseases of the respiratory system | 2 | 4 | 5 | 6 | 13 | 11 | 10 | 10 | 50 | 19 | 13 | 6 | 29 | 6 | 77 | 11 | 78 | 23 | 130 | 23 |
| K00-K93 | Diseases of the digestive system | 0 | - | 1 | 1 | 2 | 2 | 7 | 7 | 22 | 9 | 4 | 2 | 6 | 1 | 34 | 5 | 19 | 6 | 40 | 7 |
| L00-L99 | Disease of the skin and subcutaneous tissue | 0 | - | 0 | - | 0 | - | 0 | - | 0 | - | 1 | <1 | 0 | - | 8 | 1 | 2 | 1 | 10 | 2 |
| M00-M99 | Diseases of the musculoskeletal and connective tissue | 0 | - | 0 | - | 2 | 2 | 2 | 2 | 6 | 2 | 2 | 1 | 4 | 1 | 7 | 1 | 4 | 1 | 13 | 2 |
| N00-N99 | Diseases of the genitourinary system | 2 | 4 | 2 | 2 | 3 | 3 | 8 | 8 | 32 | 12 | 1 | <1 | 8 | 2 | 10 | 1 | 26 | 8 | 88 | 15 |
| O00-Q99 | Pregnancy/perinatal/congenital conditions | 2 | 4 | 3 | 4 | 1 | 1 | 1 | 1 | 2 | 1 | 4 | 2 | 0 | - | 8 | 1 | 1 | <1 | 3 | 1 |
| R00-R99 | Symptoms and signs not elsewhere classified | 1 | 2 | 4 | 5 | 4 | 4 | 4 | 4 | 6 | 2 | 15 | 6 | 17 | 4 | 23 | 3 | 13 | 4 | 5 | 1 |
| V00-Y98 | External causes of mortality | 37 | 70 | 43 | 52 | 42 | 37 | 24 | 24 | 19 | 7 | 169 | 72 | 335 | 70 | 317 | 46 | 90 | 27 | 62 | 11 |
|  | Total | 53 |  | 83 |  | 114 |  | 102 |  | 268 |  | 236 |  | 478 |  | 683 |  | 332 |  | 569 |  |

^α^mortality numbers included during the ICD-10 era, 1997-2013, ^β^for description of baseline characteristics, HBV/HCV and HBV/HCV/HIV co-infection cases were only included among people with an HBV notification

**Table S6. Adjusted analysis of factors associated with liver-related mortality among people with an HBV and HCV notification, NSW 1993-2012, n=150,403**

|  | **HBV**  **n=57,929** | | | | | **HCV**  **n=96,250** | | | | |
| --- | --- | --- | --- | --- | --- | --- | --- | --- | --- | --- |
| **Characteristics, n %** | **Deceased^α^**  **n=582** | **%** | **aHR^β, γ^** | **95% CI** | ***P*** | **Deceased^α^**  **n=2,215** | **%** | **aHR^β, γ^** | **95% CI** | ***P*** |
| **Interaction between**  **birth cohort and calendar period of viral hepatitis notification** |  |  |  |  |  |  |  |  |  |  |
| **Born ≥1965,**  **notified ≤2000** | 25 | <1 | 1.00 | - | - | 131 | 1 | 1.00 | - | - |
| **Born ≥1965,**  **notified 2001-2006** | 25 | <1 | 1.07 | 0.60, 1.89 | 0.819 | 76 | 1 | 1.02 | 0.77, 1.37 | 0.875 |
| **Born ≥1965,**  **notified ≥2007** | 16 | <1 | 1.65 | 0.85, 3.20 | 0.141 | 40 | <1 | 2.08 | 1.43, 3.02 | <0.001 |
| **Born 1945-1965,**  **notified ≤2000** | 156 | 1 | 5.64 | 3.70, 8.62 | <0.001 | 925 | 4 | 5.83 | 4.85, 7.00 | <0.001 |
| **Born 1945-1965,**  **notified 2001-2006** | 116 | 2 | 8.05 | 5.12, 12.65 | <0.001 | 438 | 4 | 9.23 | 7.50, 11.36 | <0.001 |
| **Born 1945-1965,**  **notified ≥2007** | 32 | 1 | 9.01 | 5.12, 15.88 | <0.001 | 179 | 3 | 20.92 | 16.16, 27.07 | <0.001 |
| **Born ≤1944,**  **notified ≤2000** | 135 | 5 | 28.85 | 18.72, 44.46 | <0.001 | 291 | 8 | 27.23 | 21.93, 33.82 | <0.001 |
| **Born ≤1944,**  **notified 2001-2006** | 65 | 6 | 32.09 | 19.73, 52.18 | <0.001 | 113 | 10 | 40.69 | 31.03, 53.36 | <0.001 |
| **Born ≤1944,**  **notified ≥2007** | 12 | 2 | 29.03 | 14.04, 60.04 | <0.001 | 22 | 4 | 46.66 | 29.07, 74.90 | <0.001 |

^α^records included 2002-2013, ^β^adjusted hazard ratio, **^γ^** adjusted for factors associated with liver-related mortality in multivariable analyses
